# Field and Temperature Shaping for Microwave Hyperthermia: Recent Treatment Planning Tools to Enhance SAR-Based Procedures

**DOI:** 10.3390/cancers15051560

**Published:** 2023-03-02

**Authors:** Martina T. Bevacqua, Rossella Gaffoglio, Gennaro G. Bellizzi, Marco Righero, Giorgio Giordanengo, Lorenzo Crocco, Giuseppe Vecchi, Tommaso Isernia

**Affiliations:** 1 Department of Information Engineering, Infrastructures and Sustainable Energy, Università Mediterranea di Reggio Calabria, Via Graziella, 89124 Reggio di Calabria, Italy; 2 Consorzio Nazionale Interuniversitario per le Telecomunicazioni (CNIT), Consorzio Nazionale Interuniversitario per le Telecomunicazioni, Viale G.P. Usberti, 181/A Pal.3, 43124 Parma, Italy; 3 Advanced Computing, Photonics & Electromagnetics (CPE), Fondazione LINKS, 10138 Turin, Italy; 4 National Research Council of Italy (CNR), Istituto per il Rilevamento Elettromagnetico dell’ Ambiente, CNR-IREA, Via Diocleziano 308, 80100 Napoli, Italy; 5 Department of Electronics and Telecommunications, Politecnico di Torino, 10129 Turin, Italy

**Keywords:** hyperthermia treatment planning, electromagnetic field, convex programming, field intensity shaping and focusing, electromagnetic properties

## Abstract

**Simple Summary:**

Hyperthermia is a thermal cancer treatment that consists of a selective increase in the tumor temperature to a supra-physiological value for 60–90 min. Heating via microwaves using a phased array applicator proved to be a very useful, non-invasive energy carrier. Although hyperthermia is currently employed for many anatomical sites in combination with standard techniques, there are still open challenges that prevent its more widespread use in the clinic. The aim of this article is to review the work carried out in the framework of a national project concerning the introduction of new tools for microwave hyperthermia and to unify these approaches. The proposed methodologies are interconnected and potentially allow an improvement in treatment planning using a single device.

**Abstract:**

The aim of the article is to provide a summary of the work carried out in the framework of a research project funded by the Italian Ministry of Research. The main goal of the activity was to introduce multiple tools for reliable, affordable, and high-performance microwave hyperthermia for cancer therapy. The proposed methodologies and approaches target microwave diagnostics, accurate in vivo electromagnetic parameters estimation, and improvement in treatment planning using a single device. This article provides an overview of the proposed and tested techniques and shows their complementarity and interconnection. To highlight the approach, we also present a novel combination of specific absorption rate optimization via convex programming with a temperature-based refinement method implemented to mitigate the effect of thermal boundary conditions on the final temperature map. To this purpose, numerical tests were carried out for both simple and anatomically detailed 3D scenarios for the head and neck region. These preliminary results show the potential of the combined technique and improvements in the temperature coverage of the tumor target with respect to the case wherein no refinement is adopted.

## 1. Introduction

Hyperthermia (HT) is a thermal cancer treatment that consists of a selective increase in the tumor temperature to a supra-physiological value (40–44 °C) for from 60 to 90 min. Clinical trials have demonstrated the therapeutic benefit of this treatment in combination with radiotherapy and chemotherapy [1,2,3,4,5]. A plethora of different thermal techniques and applicators have been investigated over the years to efficiently transfer heat to the body in medicine [6]. Heating via microwaves using a phased array applicator proved to be a very useful, non-invasive energy carrier [6,7].

Although hyperthermia is in clinical use for many anatomical sites, there are still open challenges. First, the accuracy of hyperthermia treatment planning (HTP) depends on the accuracy with which electromagnetic (EM) and thermal tissue parameters are known. Second, a tool able to provide accurate real-time monitoring of the temperature distribution administered to the patient is required, and it is still not available. Currently, magnetic resonance-based approaches exist [8,9]; however, widespread use in the clinic is hampered by the inaccuracies introduced by movements [9] (e.g., breathing, bowels, blood vessels), the challenges faced when applying treatments inside a hybrid HT-MRI system, and the high cost. 

The above-reported issues concerning HT are crucial, especially for challenging anatomical sites such as the head and neck (H&N) region and when real-time feedback control is absent, and thermo-regulation mechanisms are unknown [6,10,11].

HTP involves obtaining the optimal complex excitation coefficients (phases and amplitudes) of the signals feeding a microwave-phased array applicator. The objective of this procedure is to induce a homogeneous temperature increase in a given target area while avoiding high temperatures in healthy tissues (i.e., hotspots). 

Once the 3D patient model has been segmented and tissue properties assigned, the HTP flow includes the evaluation of the EM fields in the domain of interest and, hence, the optimization of the phases and amplitudes feeding the applicator. Whether the specific absorption rate (SAR) or temperature distribution should be optimized is still a topic of debate amongst hyperthermia researchers [10,11,12,13], and both SAR-based and temperature-based (T-based) optimizations are currently successfully used in the clinic providing comparable results [14].

Optimizing the temperature distribution would seem the most natural approach since the increasing temperature is obviously the basis for obtaining therapeutic effectiveness in hyperthermia treatment. Moreover, systematic studies to assess the actual relation between the predicted temperature and the clinical outcomes have been proposed [14,15,16]. However, T-based optimizations require global optimizers, which are generally affected by a high computational cost and problem-specific parameter tuning [17].

Conversely, SAR is directly related to the complex excitation coefficients via Maxwell’s equations. Additionally, studies, such as [18], demonstrated that a relation exists between SAR coverage indicators and clinical outcomes of hyperthermia treatments for different anatomical sites and applicators. Further, SAR can be experimentally validated within a quality assurance procedure. Hence, the SAR performance of a hyperthermia system can be computationally and metrologically controlled [10]. Whatever the case, while results in contrast with the one in [19] have been found by de Greef et al. [20], optimizing the SAR pattern is faster than optimizing the temperature profile since the former may be formulated as a convex optimization problem (COP) with respect to the unknown feedings and does not require solving the bioheat equation [12].

Because of the attractive features recalled above, many SAR-based optimization strategies have been proposed in the literature. Some approaches aim at maximizing the SAR within the target volume while minimizing it in the surrounding healthy tissues. In some cases, also global optimizers have been used [21,22]. In [23,24], HTP optimization routines based on the well-known time-reversal (TR) approach [25] have been proposed. While very intuitive, straightforward, and with a negligible computational time, the classic TR approach suffers from two main drawbacks [25]: the need for the so-called TR mirror (i.e., a source surrounding the region under test) and the impossibility of controlling hotspots. Some efforts to overcome these two issues have been made recently in the literature [26,27]. Additionally, quadratic approaches to SAR optimization have been proposed [28,29,30].

The optimization of the phases and amplitudes feeding the applicator depends on the adopted 3D EM patient model. One of the main limitations of HTP is the absence of accurate, reliable, and robust knowledge of the in vivo EM and thermal parameters of the different tissues. Currently, trained clinicians create 3D patient models by segmenting patient scans obtained via magnetic resonance imaging (MRI) or computed tomography (CT). Then, the corresponding parameters are associated with each tissue based on the average and ex vivo data [6,31]. In such a way, the EM property distributions are approximated with a step-wise constant function. This is a common assumption when therapeutic treatments are planned, or field exposure is quantified [32,33]. However, for what concerning ex vivo EM properties, there is no consensus on their accuracy with respect to in vivo values. Many studies have shown a not negligible difference existing between ex vivo and in vivo EM parameters (EPs) [34,35,36]. Moreover, ex vivo parameters do not account for dissimilarities among individuals. In HTP, these differences may easily result in unpredicted hotspots in a patient and an actual SAR distribution different from the desired one.

Another limitation is that a proper SAR shaping in a realistic scenario cannot correspond by default to the desired temperature distribution due to the thermal boundary conditions arising from external cooling systems (waterbolus) and physiology (e.g., the air flow in respiratory tracts) [37,38].

**Figure 1:**
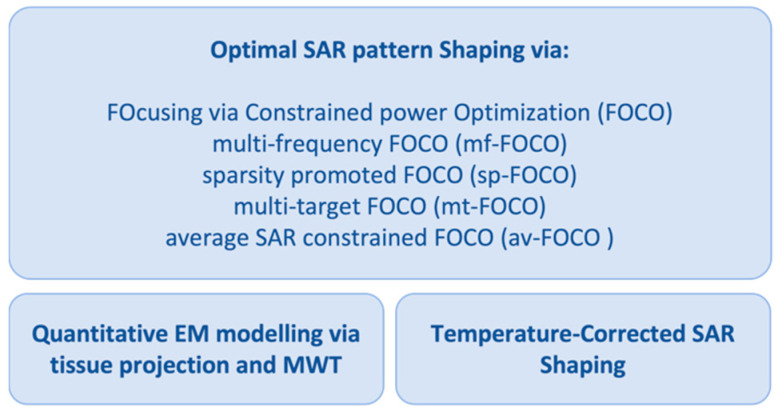
Schematic diagram of the reviewed research activities to advance HTP.

This paper aims to review and present, in a unified fashion, the work carried out by the authors along several concurrent directions to advance hyperthermia; the framework for these research lines was a national project. Advancements have been pursued in the algorithms for optimal SAR shaping based on convex optimization (as opposed to global optimization via meta-heuristics) and the approach called “FOcusing via Constrained power Optimization” (FOCO). Complementary procedures have also been proposed to mitigate variations of SAR and temperature distributions due to the ex vivo (rather than in vivo) EM modeling of the electromagnetic scenario and thermal boundary conditions (see Figure 1). The impact of the quantitative EM modeling on the use of FOCO for SAR pattern shaping has been studied in [39]. Here, the FOCO SAR shaping algorithm and the strategy for Temperature-Corrected SAR shaping are combined and tested for the first time against two 3D numerical scenarios mimicking the neck region in order to show the strong interconnections of the research activities carried out by the authors and highlight the links between the proposed tools.

## 2. Materials and Methods

### 2.1. Optimal SAR Pattern Shaping

The innovative contribution of the research activities reported here was centered around a novel optimization framework for the optimal shaping of a wavefield, a relevant problem in many different applications.

In particular, the reviewed activities led to the introduction of a novel shaping paradigm based on convex programming and on the approach called “FOcusing via Constrained power Optimization”. This latter was first proposed in the antenna framework for the optimal synthesis of pencil beams [40] and then extended to HTP [12]. FOCO aims at focusing the SAR distribution in the target volume and, at the same time enforcing hotspot-limiting constraints in the healthy tissues. 

**Figure 2:**
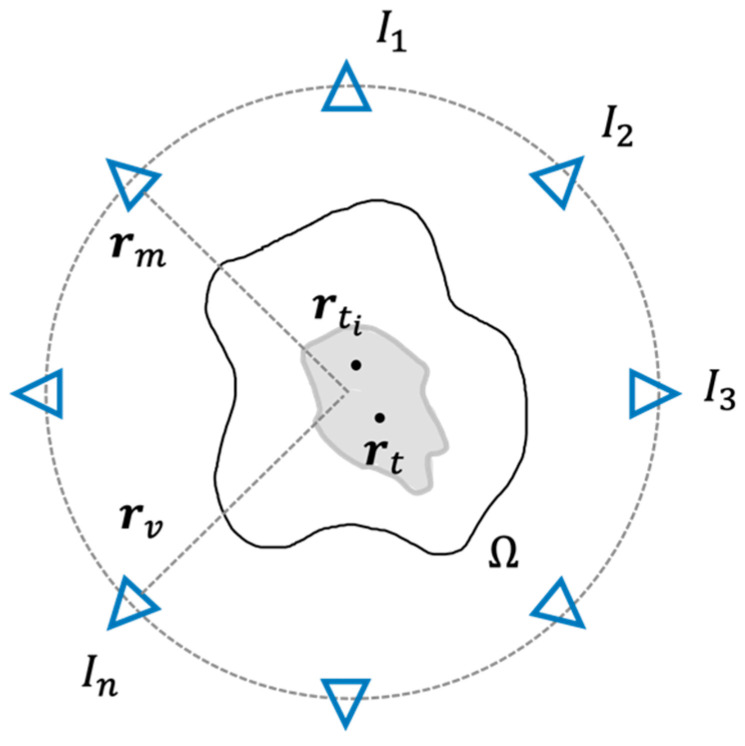
Schematic view of the phased antenna array surrounding the domain of interest Ω and the target volume. Each antenna is indicated by a blue triangle, while the grey area represents the target volume.

From a mathematical standpoint, if we consider a target point properly set within the target area identifying the tumor (the grey area in Figure 2), say $\;r_{t}\in \Omega$, a generic constrained focusing problem could be stated as: 

*Determine the set of the array’s complex excitation coefficients such to maximize the squared amplitude of the field in the target point* $r_{t}$ *, while enforcing arbitrary upper bounds in the rest of the domain of interest.*

Unfortunately, this maximization problem is non-linear and belongs to the class of NP-hard problems [17]. 

#### 2.1.1. Description of FOCO and Derived Approaches

To circumvent the non-convexity of the problem, FOCO exploits the degree of freedom of the field phase reference and assumes the field components $E_{i}\left(r\right)$ dominant above the other ones and real in the target point [12,40]. Then, the problem can be stated as: 

Find $I_{n}\;\left(n=1,\ldots ,\;N_{A}\right)$ such to:

(1a) $$\max ℜ\left\{E_{i}\left(r_{t}\right)\right\}$$

subject to:

(1b) $$ℑ\left\{E_{i}\left(r_{t}\right)\right\}=0$$

(1c) $${\left|E\left(r\right)\right|}^{2}\;\leq \;\;M\left(r\right)\;\;\;\;\;\;r\in \Omega \backslash \Pi \left(r_{t}\right)$$

where $r=\left(x,y,z\right)$ scans the domain Ω, E is the total field and can be expressed as the superposition by the complex excitation coefficients $I_{n}$ (feeding the $N_{A}$ antennas composing the applicator) of the total electric fields induced by each unitary excited antenna when all the other antennas are off [12]. $ℜ\left\{\cdot \right\}$ and $ℑ\left\{\cdot \right\}$ represent the real part and the imaginary part, respectively. 

$M\left(r\right)$ is the mask function, which is a non-negative arbitrary function that allows enforcing patient-specific constraints on the power deposition outside the chosen target volume $\Pi \left(r_{t}\right)$. Higher weights are generally applied to tissues exhibiting higher power losses to counteract undesired heating. Considering the physical limitation of the focusing capability of any phased array applicator in tissues [41], this target volume $\Pi \left(r_{t}\right)$ can be assumed to be a sphere with diameter $\lambda_{m}/3$ , being  $\lambda_{m}$ the shortest wavelength among those of the different tissues.

Being cast as a convex programming problem, FOCO does not require global search algorithms and it delivers the globally optimal solution regardless of problem size (i.e., number of unknowns/antennas) or parameters tuning. Unlike the approach in [29], which adopts a semidefinite relaxation, FOCO does not consider any kind of approximations and simply exploits the degree of freedom on the field phase reference. Moreover, FOCO does not require an additional procedure for the final retrieval of the excitation vectors. 

Starting from the above, four constrained approaches have been proposed, aimed at focusing a wavefield in a target point and based on FOCO. Specifically, these latter are:

- Multi-frequency FOCO (mf-FOCO) [42], based on the idea that hotspot spatial collocations could change with frequency. Hence, by exploiting such a feature and adopting multi-frequency applicators, one could alleviate hotspots occurrence (or mitigate their impact).
- Sparsity promoted FOCO (sp-FOCO) [43], introduced to address the need to optimally select the active elements of a given applicator in a patient-specific fashion. From a mathematical point of view, it implies in problem (1) the presence of a constraint in ${𝓵}_{1}$-norm, borrowed from the compressive sensing theory [44], that is:
  (1d) $$∥I_{n}∥{}_{{𝓵}_{1}}\leq \delta$$
  wherein δ is a tunable parameter. The above constraint promotes the sparsity of the solution and, hence, allows to reduce the number of active elements in the array configuration that is able to maximize the SAR within the target volume and to avoid undesired heating in healthy tissues at the same time.
- Multi-target FOCO (mt-FOCO) [45], aiming at uniformly shaping the SAR over an extended target area that may have irregular contours (i.e., late-stage tumors). Nowadays, this task is not efficiently addressed by the clinically adopted algorithms. From a mathematical point of view, it involves two additional constraints, that are:
  (1e) $$R\left\{E_{i}\left(r_{t_{i}},I_{n}\right)\right\}=ℜ\left\{E_{i}\left(r_{t},I_{n}\right)\right\}\cos\phi_{i}$$
  (1f) $$ℑ\left\{E_{i}\left(r_{t_{i}},I_{n}\right)\right\}=ℜ\left\{E_{i}\left(r_{t},I_{n}\right)\right\}\sin\phi_{i}$$
  wherein $r_{t_{i}}\;\left(i\;=1,\ldots ,L\right)$ is a set of control points located in the chosen target area and $\phi_{i}\in \left[0,2\pi \right]$ are the auxiliary variables indicating the phase shifts between the field in $r_{t}$ and $r_{t_{i}}$. The above constraints guarantee the uniformity of the field in the target region. For any fixed value of $\phi_{i}$, the problem is cast as the maximization of a linear function in a convex set, which corresponds to a COP. As such, mt-FOCO is able to determine the globally optimal solution.
- Average SAR-constrained FOCO (av-FOCO) [46], which enforces hotspot-preventing constraints on the average SAR distribution rather than on the voxel-vise SAR. This is related to the fact that the average SAR over IEEE peak SAR quantifiers (1 g, 10 g) [47] is physically more related to temperature rather than the punctual SAR, i.e., voxel-vise [48].

All the above FOCO-based procedures can be extended to the case of vectorial fields, as discussed in [49].

#### 2.1.2. Assessment of FOCO-Based Approaches against Clinical Data

Some of the above strategies have been clinically tested and comparatively assessed on actual patient data in collaboration with the Hyperthermia Unit of the Department of Radiation Oncology at the Erasmus MC (Rotterdam, The Netherlands) [43,46,50,51]. The clinical assessment has been pursued within the very challenging clinical scenario of patients with H&N cancer. This scenario presents a very good case for the assessment of the benefit of novel shaping approaches. In fact, target conformal heating is possible, and therefore the optimal planning of the treatment is pivotal and routinely used. Therefore, this scenario is highly suited to our aims in terms of predicted treatment quality and computational costs.

For instance, in [50], FOCO is analyzed in a clinical scenario and compared to the VEDO approach [48]. VEDO tackles the planning in hyperthermia treatment as a multi-objective optimization problem. In particular, VEDO maximizes the SAR within the target volume while minimizing it in the hotspots. Due to the above, the relevant cost function, that is, the Target to Hotspot SAR Quotient (THQ), is defined as the ratio between the mean SAR in the target region and the average SAR in the hotspots (which is the 1% of the healthy volume with the highest SAR) [48]. Unlike FOCO, this optimization problem is non-convex and is usually performed with the Particle Swarm Optimization (PSO) algorithm. The results achieved in [50] show that FOCO performs comparably to the clinical benchmark overall (ΔT50 = +0.05 °C) but outperforms the benchmark on average for target volumes above approximately 40 cm^3^ (ΔT50 = +0.39 °C). In addition, being FOCO formulated as a convex optimization problem, the results of the optimization procedure are repeatable and achieved sensibly faster (−44%) [50]. 

It should be noted that the final thermal distribution obtained with the optimized feedings may be different from the desired one in a realistic scenario due to both the inaccuracies in the ex vivo EM patient model and the thermal boundary conditions (that are not considered in the SAR optimization). To circumvent such issues, two complementary strategies, which can be used singularly or eventually in combination, have been proposed, as presented in the following sections.

### 2.2. Refinement of SAR Planning via Microwave Tomography Based Quantitative EM Modelling

As already stated in the Introduction, the final SAR map (and the corresponding thermal distribution) obtained with the optimized antenna feedings may be different from the actual (desired) one due to the inaccuracies introduced by the ex vivo EM modeling of the patient. By “quantitative modeling of the scenario,” we mean the process of obtaining the parameters of the EM model of the anatomical region to be treated. This is essential not only for accurate patient-specific delivery of the thermal treatment but also for the prediction of the EM field distribution inside the body in many biomedical applications, from safety assessment of medical devices to dosimetry and radioprotection studies. 

The present state of the art is based on the segmentation of medical images (MRI/CT) and the use of a conventional database of EM and thermal properties of tissues from ex vivo measurements [31]. In this respect, in literature, many efforts have been pursued to introduce innovative techniques for non-invasively measuring the in vivo properties. 

The first approach consists in processing the MRI data corresponding to different arrangements of the coils at the resonance frequency [52,53]. As useful radiofrequency data can be collected inside the region of interest, such an inverse problem is affected by reduced ill-posedness with respect to more usual inverse scattering problems [54,55]. Then, a spatial resolution in the order of a few millimeters and high accuracy can be achieved in the determination of the complex permittivity of the scenario. However, such retrieval is performed at the resonance frequency of the MRI apparatus, and hence, a reliable dispersion relationship must be assumed to extrapolate data to the frequencies of interest in hyperthermia. Moreover, uncertainties may be present in phase measurements of useful radiofrequency data [53]. 

In this respect, the research activities by the authors of [39] have resulted in the development of an alternative, innovative, and low-cost approach based on microwave tomography (MWT) [54]. MWT is a low-cost and non-invasive modality to image the inside of regions not directly accessible. MWT can be an excellent imaging candidate, as it allows to retrieve the patient-specific and actual in vivo EM properties of the tissues to be thermally treated. However, with respect to the current state of the art, which exploits MRI and/or CT, MWT exhibits a low spatial resolution, which would ultimately impair the final in vivo estimation. 

#### Description of the Proposed Segmented MWT

To counteract the resolution issue, an innovative inversion approach has been developed in [39], which incorporates the morphological information from the existing segmentation of the (pre-treatment) MRI or CT images into the MWT algorithm. In this way, the EPs distributions are approximated with step-wise constant functions, which is a common assumption in HTP. Moreover, the underlying inverse scattering problem [56] is dealt with as a parameter estimation technique where a single (complex) parameter is looked for in each different sub-region of the domain under test. This alleviates the well-known difficulties associated with generic inverse scattering problems [56] since the strongly reduced number of parameters results in a strongly reduced ill-posedness and non-linearity. Opposite to MRI-based tomography, this approach will allow retrieving the effective patient-specific EM parameters of interest at the frequencies used in hyperthermia.

The proposed patient-specific representation basis, in the following denoted as “tissue projection”, has been used in conjunction with the well-known contrast source inversion (CSI) scheme [57] but can also be combined with other inversion schemes. From a mathematical point of view, the problem of quantitatively understanding the EM scenario is cast as the minimization of the following non-linear cost functional:

(2) $$\underset{\chi ,W}{\min}\underset{v}{\sum }\frac{∥T\left(\hat{\chi }\right)E_{inc}+T\left(\hat{\chi }\right)A_{i}\left[W\right]-W∥{}_{{𝓵}_{2}}^{2}}{∥E_{inc}∥{}_{{𝓵}_{2}}^{2}}+\underset{v}{\sum }\frac{∥E_{scat}-A_{e}\left[W\right]∥{}_{{𝓵}_{2}}^{2}}{∥E_{scat}∥{}_{{𝓵}_{2}}^{2}}$$

wherein ${\left\|\cdot \right\|}_{{𝓵}_{2}}$ denotes the ${𝓵}_{2}$-norm, $E_{inc}\left(r,r_{v}\right)$, $E_{scat}\left(r_{m},\;r_{v}\right)$ and $W\left(r,r_{v}\right)$ are the incident field, the scattered field and the contrast source induced in the scatterers, respectively. $r_{v}$ and $\;r_{m}$ identify the positions of the transmitting and receiving antennas surrounding Ω, as shown in Figure 2. $\;A_{e}$ and $A_{i}$ are short notations for the corresponding integral radiation operators. Finally, $\hat{\chi }$ are the unknown effective electrical properties corresponding to each tissue, while T is the inverse of the operator, which projects the electrical properties of the tissue from the pixel basis to the tissue one. In particular, this latter is composed of a set of functions, such that the generic tissue basis function is different from zero in all pixels associated with the n-th tissue and zero elsewhere. 

The proposed estimation problem amounts to recovering the effective EM properties of tissues ${\hat{\chi }}_{1},\ldots ,{\hat{\chi }}_{N}$ starting from the knowledge of the incident fields $E_{inc}$ and the measurements of the corresponding scattered fields $E_{scat}$. More details as well as an assessment of 2D scalar problems (TM polarized fields) can be found in [39]. Note that the above tissue projection belongs to the hard prior regularization techniques [58,59].

From a technological point of view, the feasibility of the proposed approach relies on dual use of the existing antennas both to deliver a hyperthermia treatment as well as to retrieve in vivo tissues EM parameters. In particular, a multiview-multistatic measurements configuration is suggested, wherein the antennas are organized in such a way that for each transmitting antenna in $r_{v}$ all the antennas in $r_{m}$ act as receivers, and all antennas alternately act as a transmitter. However, the approach can be extended and tailored in case of simpler measurement configurations. Indeed, although phase measurements are, of course, possible at microwave frequencies, a significant reduction of the complexity of the required hardware and the related cost can be obtained if only amplitude data are collected. Moreover, simpler MWT devices are more easily integrable within existing clinical systems in hyperthermia treatments. For this reason, in [60], the proposed estimation technique has been extended and tested against only amplitude data.

Finally, the tissue projection approach is affected by the capability of co-registering MRI and CT images with the MWT system. This is a challenging issue. However, some devices can already circumvent this difficulty in the hyperthermia treatment of H&N tumors [22].

### 2.3. Temperature-Corrected SAR Shaping

Although it is well known that in a homogeneous infinite medium, the SAR and the corresponding temperature map have the same peak locations [12], in a realistic scenario, a proper SAR focusing does not correspond by default to the desired temperature distribution, due to the thermal boundary conditions arising from external cooling systems (waterbolus) and physiology (e.g., the air flow in respiratory tracts) [37]. 

In [38], we have analyzed this aspect from a numerical point of view, and we have proposed an easy-to-implement strategy aimed at finding the optimal shift in the SAR focusing that mitigates the effects of the boundary conditions, maximizing the temperature rise in the tumor region. The presented approach has been formulated to improve the performance of SAR-based optimization routines, such as VEDO [48] or the previously described FOCO technique [12,50].

#### 2.3.1. Description of the T-Correction Approach

The temperature correction approach can be generally described by means of the steps reported below.

1. Following standard HTP procedures, a SAR-based optimization is performed to maximize the power deposition on the tumor target region (centered at $r_{t}$), minimizing the risk of hotspots in the surrounding heathy tissues.
2. The optimized squared magnitude of the electric field is reasonably approximated by a (multi-variate) Gaussian fitting function, with different standard deviations along the different axes and peak position $r_{0}$.
3. The peak position $r_{0}$ of the Gaussian fitting function is moved in a refinement region $V_{R}$ defined around the tumor target, where a proper number of points ($N_{RFN}$) is considered.
4. For each point in the refinement region $V_{R}$, the Gaussian fitting function is used as the source term of the bioheat equation, and the following fitness function is computed:
  (3) $$\tau_{90}=\frac{T90}{\max_{r\in V_{T}}\left\{T\left(r\right)\right\}}$$
  where the T90 parameter [61] is defined as the temperature exceeded by 90% of the points in the tumor region $V_{T}$, while power has been increased in the temperature simulations until the maximum temperature in normal tissue reached 43 °C. The fitness function reported in (3) has been formulated to provide both a good temperature focusing on the tumor and a more uniform temperature coverage of this region. It should be noted that the $\tau_{90}$ parameter is a surrogate parameter, defined in the context of our numerical tests, with no correlation with the clinical outcome.
5. The center $r_{0}={\tilde{r}}_{t}$ corresponding to the maximum value of $\tau_{90}$ provides the shifted focusing center for a new SAR-based optimization, able to provide an improved temperature coverage of the tumor region $V_{T};$
6. Point 1 is repeated to optimize the SAR on a target region centered around ${\tilde{r}}_{t}$.

As emphasized in [38], the proposed approach has been formulated to obtain a refinement in the temperature focusing by solving the bioheat equation a limited number of times (i.e., not including it in a global optimization routine).

In the next section, the optimization implemented by FOCO (described in Section 2.1) and the above-described thermal refinement procedure are preliminarily combined and applied to both a simplified and a realistic 3D numerical testbed, reproducing the microwave heating of a tumor placed in the human neck. Differently from [38], wherein a global particle swarm optimization of the target-to-hotspot SAR quotient (THQ) [22] was adopted, the use of FOCO allows a significantly lower computational cost.

## 3. Results

### 3.1. D Numerical Scenario

Two numerical scenarios were considered: a simplified model of the human neck and an anatomically detailed H&N model.

**Figure 3:**
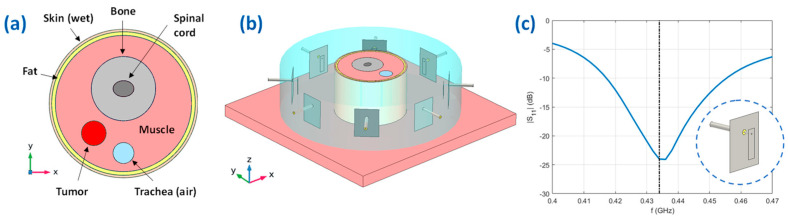
Simplified numerical testbed. (**a**) Top view on the plane $z=z_{t}=-15$ mm of the simple neck model implemented in COMSOL, with the considered different tissues; (**b**) Geometry of the HTP applicator, the simplified neck, and the waterbolus; (**c**) Reflection coefficient of a single-feed patch antenna of the simulated array.

**Figure 4:**
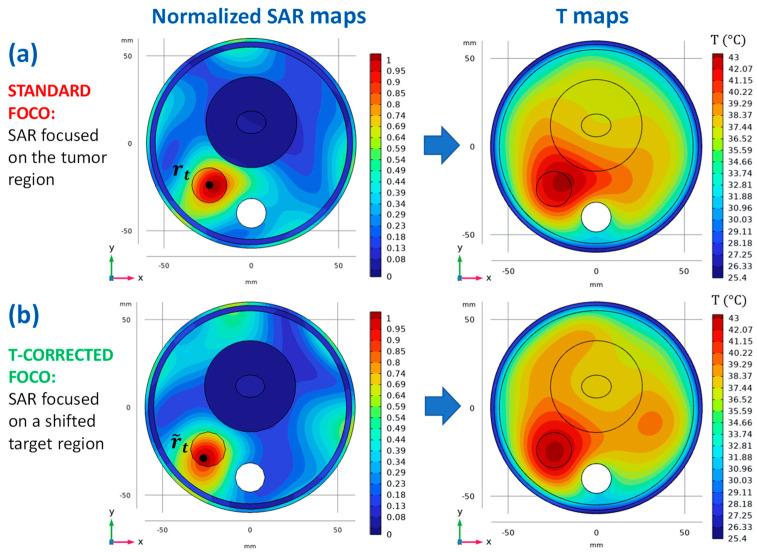
SAR and temperature maps for the simplified testbed. (**a**) Normalized SAR map, visualized on the plane z=−15 mm, optimized with FOCO for a target tumor centered around its centroid, at $r_{t}=\left(-24,-24,-15\right)$ mm (left) and the corresponding temperature map (right); (**b**) Normalized SAR map, visualized on the plane z=−15 mm, optimized with FOCO for a target region centered around the corrected center ${\tilde{r}}_{t}=\left(-26.7,-29.8,-15\right)$ mm (left) and the corresponding temperature map (right).

**Figure 5:**
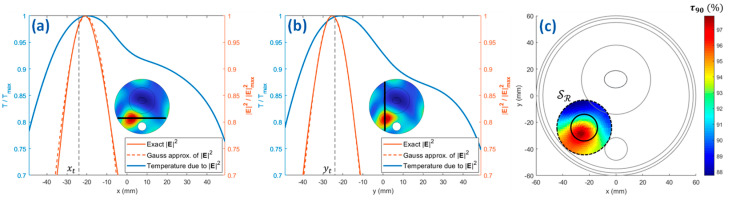
SAR Gaussian approximation for the simplified testbed. Squared amplitudes of the electric field norm (see the insets) along the x (**a**) and y (**b**) axes passing through the tumor centroid, their Gaussian approximations and the temperature profiles corresponding to the exact fields, after the first FOCO optimization (no correction is introduced); (**c**) fitness function $\tau_{90}$ as a function of the Gaussian SAR focusing center on the two-dimensional refinement region $S_{R}$ (dashed circle). The boundary of the tumor region on the plane z=−15  mm is highlighted by a solid circle.

The simplified numerical testbed was modeled in COMSOL Multiphysics [62] and is reported in Figure 3, Figure 4 and Figure 5. The phantom region is a simplified version of the human neck, where the trachea, the vertebrae, and the neck shape are modeled as cylinders. The tumor is represented by an ellipsoid centered at $r_{t}=\left(-24,-24,-15\right)$ mm, with semi-minor axes $a_{x}=a_{y}=10$ mm and semi-major axis $a_{z}=15$ mm. The dielectric and thermal parameters have been assigned to the different tissues according to [63,64] and [65] for the tumor at the operating frequency f=434 MHz. The applicator is a phased circular array made of N=8 patch antennas immersed in the waterbolus, properly optimized to resonate at 434 MHz with a sufficiently large bandwidth of about 20 MHz at −15 dB (see the reflection coefficient reported in Figure 3c, obtained when only one antenna is fed, while all the others are switched off). The length and the width of the optimized patch elements are 28.75 and 8.41 mm, respectively, while the distance from the ground is 8.81 mm. As shown in Figure 3b, the neck cylinder is positioned on the top of an absorbing flat muscle layer, which mimics the human shoulders and decreases the influence of the water-air transition [66].

**Figure 6:**
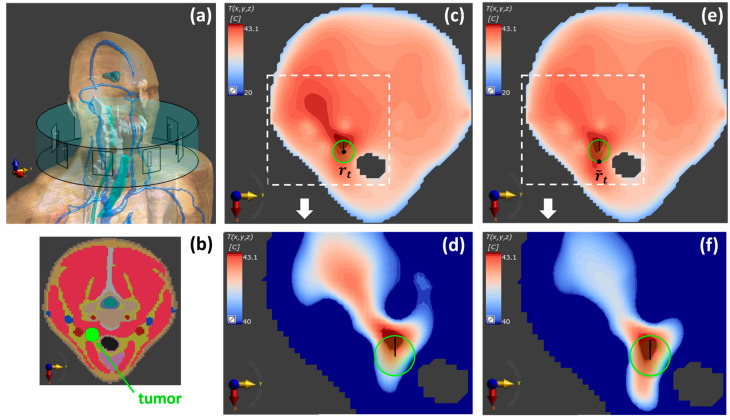
Temperature maps for the realistic testbed. (**a**) Numerical model implemented in Sim4Life with the realistic phantom Duke [67]. (**b**) Segmented tissues on the $z=z_{t}$ plane. Temperature maps on the $z=z_{t}$ plane corresponding to a SAR map optimized with FOCO (**c**,**d**) for a target region centered around the tumor centroid $r_{t}$ and (**e**,**f**) for a target region centered around the corrected center ${\tilde{r}}_{t}$. (**d**,**f**) magnify the region around the tumor target of (**c**,**e**), respectively, with an expanded color scale. The boundary of the tumor region is highlighted by a solid green circle. From (**c**–**e**), an improvement of the temperature coverage is observed, as well as a significant hotspot suppression.

The anatomically detailed scenario was implemented by using the simulation software Sim4Life V6.2.2 (Zurich Med Tech AG, Switzerland) and the realistic human phantom Duke V3.0 [67] (see Figure 6. In this case, the tumor was modeled as an ellipsoid with $a_{x}=a_{y}=7$ mm, $a_{z}=9$ mm, and tissue properties as defined in [65]. The main characteristics of the considered array of patch antennas are the same as those described above for the simplified numerical scenario.

In both models, the thermal boundary conditions were assigned according to [38], with convective heat flux boundary conditions introduced on the skin-waterbolus interface and on the internal boundaries of the trachea. Moreover, it is important to note that while for the case of the simplified model, all the steps reported in Section 2.3.1 were implemented, for the realistic model, we decided to replace the Gaussian approximation (*step 2*) with multiple SAR optimizations, which is a choice made possible by the low computational cost of a convex optimization approach such as FOCO.

### 3.2. Numerical Proof-of-Concept

We refer to the steps reported in Section 2.3.1 and to Figure 4, Figure 5 and Figure 6 while describing the application of the temperature-corrected FOCO technique to the numerical models illustrated in Section 3.1. The SAR and temperature distributions obtained with the standard FOCO are also shown for the sake of comparison.

For the simplified numerical scenario, as a first step, FOCO was applied to optimize the field focusing on the tumor target, centered at $r_{t}=\left(-24,-24,-15\right)$ mm (*step 1*). We have considered the z component as the dominant one, as indicated in [50]. 

Figure 4a shows the resulting normalized SAR map, on the plane z=−15 mm and the corresponding temperature profile obtained by using the optimized SAR as the source term of the bioheat equation and the boundary conditions described in [38]. As can be noted, a significant temperature shift outside the tumor region occurs due to the thermal effects introduced by the waterbolus and the air flux in the trachea.

To correct this shift, the squared amplitude of the optimized E-field (corresponding to the SAR distribution of Figure 4a) was fitted with a two-dimensional Gaussian mask with amplitude $a=1.39\times 10^{5}$ V^2^/m^2^ and standard deviations $\sigma_{x}=18$ mm and $\sigma_{y}=16.5$ mm (see Figure 5a,b) (*step 2*). In the reported example, we limited the refinement region (*step 3*) to a two-dimensional circle $S_{R}$ defined on the plane z=−15 mm and centered around $r_{t}$. A reasonable diameter for $S_{R}$ is suggested to be: $d_{R}=2\left(a_{x}+\Delta \right)=38$ mm, being Δ the main distance between the SAR and temperature peaks (see Figure 5c) [38]. Then, the center of the Gaussian mask has been moved on $N_{rFN}=\;$491 centers $\left(x_{0},y_{0}\right)\;$ equally spaced on $S_{R}$, for which a 2D version of the bioheat equation has been solved. The considered value for $N_{rFN}$ is definitely an overkill; the scale length of the spatial discretization for the refinement is reasonably given by the shift Δ between the SAR and temperature maps, which in typical cases leads to a much smaller number of refinement points. The resulting $\tau_{90}$ parameter (3) computed for each point in $S_{R}$ is reported in Figure 5c (*step 4*), and the maximum value was found for $\left({\tilde{x}}_{0},{\tilde{y}}_{0}\right)=\left(-26.7,-29.8\right)$ mm (*step 5*). Finally, FOCO was again applied to focus the EM radiation on a target region centered around the shifted position ${\tilde{r}}_{t}=\left({\tilde{x}}_{0},{\tilde{y}}_{0},-15\;\mathrm{mm}\right)$ $=\left(-26.7,-29.8,-15\right)$ mm (*step 6*).

Figure 4b shows both the optimized SAR profile and the corresponding temperature map after the application of the thermal refinement procedure.

A comparison between FOCO and a global particle swarm optimization (PSO) of the THQ [22,38] is reported in Table 1 in terms of the temperature indices T50, T90 [61], and $\tau_{90}$.

Regarding the realistic testbed, FOCO was first applied to optimize the SAR distribution on the tumor target centered at $r_{t}=\left(157,\;247,\;1565\right)$ mm (*step 1*). The corresponding temperature map is reported in Figure 6b, showing a profile that is not optimally centered on the tumor target. Because of the very low computational cost of FOCO, we implemented the search of the optimal shifted SAR focusing center ${\tilde{r}}_{t}$ by performing multiple FOCO optimizations for different candidate positions; the latter were considered placed on a 2D circular refinement region $S_{R}\;$ centered around $r_{t}$ on the $z=z_{t}$ plane, with diameter $d_{R}=30$ mm. The reference distance used to choose the points on $S_{R}\;$ was dictated by the shift Δ≈7 mm observed in Figure 6b, between the tumor center and the temperature peak position; this resulted in $N_{rFN}=\;$ 6. For each FOCO SAR optimization, the $\tau_{90}$ parameter was evaluated (*step 4*) and the optimal focusing point determined by the position maximizing the $\tau_{90}$; this resulted into ${\tilde{r}}_{t}=\left(164,\;247,\;1565\right)$ mm (*step 5*) and led to the optimized temperature map reported in Figure 6c. 

## 4. Discussion

The above preliminary examples aim at showing the interconnection between two of the tools proposed in the research initiative here reviewed (i.e., FOCO and the T-corrected SAR shaping) and not at assessing the benefits of the resulting T-corrected FOCO and at comparing it with other state-of-the-art approaches. As can be noted in Figure 4 and Figure 6, after the correction, an improvement in the temperature coverage of the tumor target has been achieved with respect to the standard FOCO. Moreover, the temperature peak appears more centered on the tumor. This is confirmed by an increase in the $\tau_{90}$ parameter in the tumor region from 95% to 97% for the simplified testbed (Table 1) and from 94% to 95.2% for the realistic testbed before and after the application of the thermal refinement procedure. Note that for the realistic case, a slightly denser discretization of the refinement region is expected to lead to further improved tumor temperature coverage. 

As discussed in [38], the computational burden of direct temperature optimization has been calculated to be higher than that of a SAR-based optimization with the proposed thermal refinement procedure. Moreover, the use of FOCO further reduces the computational cost of the proposed combined technique with respect to [38], where the global particle swarm optimization of the THQ is considered, with comparable results (see Table 1). The advantage of FOCO versus a global optimization is especially evident in the case of multiple SAR optimizations (herein implemented in the realistic scenario), leading to running times of minutes in place of hours. Indeed, for the realistic case, the computational burden of FOCO requires a running time of about 1 min, while the one related to global optimization requires a running time of about half an hour. 

Regarding the clinical implications of the overall activities herein reviewed, some considerations are in order. First, FOCO has been demonstrated to have comparable performance to the current clinical HTP methodologies [50]. In the same comparison, FOCO has been shown to deliver better results in the case of larger target volumes (>40 cm^3^). Lastly, FOCO-based procedures are formulated as a convex optimization problem, hence, are easier to be implemented (as no tuning of parameters is required) and much faster than PSO-based methods. As opposed to non-convex optimizers, the results are then more repeatable and computationally less cumbersome (see above). Moreover, the point target correction based on temperature analysis could further improve FOCO performance with respect to the standard FOCO and make more effective the corresponding thermal treatment. 

Second, as far as measurement-based quantitative EM modeling is concerned, the knowledge of EM properties is limited to ex vivo or animal studies with limitations on the knowledge of the dependencies to temperature and intra- and inter-patient variability. Solving this fundamental issue will contribute to the plan-of-the-day approaches to tackling undesired hotspots. 

Finally, a common trait of the research line reviewed here has been the effort to move from diagnostic or therapeutic devices into theranostic devices. The capabilities of the developed techniques enable, in principle, the possibility of using the same medical device for both diagnostic and therapeutic purposes with non-negligent economic impact of the adoption of MW devices.

## 5. Conclusions

In this paper, the review of a set of research lines carried out by the authors in the field of microwave cancer hyperthermia has been presented, emphasizing their complementarity and their interconnections. The research activities have been devoted to the development of new tools to increase the effectiveness and patient-specificity of hyperthermia treatments. The crucial issue of optimal design of the array applicator has been addressed. Moreover, two complementary techniques to further optimize the SAR and temperature distributions and ensure an improved temperature coverage of the target area have been developed. The first one relies on a quantitative assessment of the EM scenario via MWT, while the second one takes into account the thermal boundary conditions. They can be used individually or in combination.

Finally, the proposed SAR optimization convex-programming procedure (FOCO) has been combined for the first time with the refinement technique proposed in Section 2.3. The preliminary tests against numerical scenarios mimicking a human neck show promising results. 

Future works will be aimed at testing the overall proposed strategies (including the one for quantitative modeling of the EM scenario) against the ESHO patient repository [68]. In particular, a comparison to HTPs obtained without MWT (i.e., using tissue properties from literature) will be performed. 

Moreover, the case of the extended tumoral region will be addressed by considering the shaping paradigm underlying multi-control points-based approaches [45,69] and by extending the thermal refinement procedure. 

Finally, T-corrected FOCO with the Gaussian approximation (or even multiple SAR optimizations) has shown a computational cost that allows sensitivity studies with respect to critical parameters such as perfusion, which are crucial for the applicability of the method in a clinical setting.

## Figures and Tables

**Table 1:** T50, T90, and $\tau_{90}$ parameters for the simplified testbed (with maximal normal tissue temperature of 43 °C), corresponding to a SAR-based optimization implemented with FOCO and with a global optimization of the THQ. Pre and post prefixes refer to the temperature map before and after the application of the thermal refinement procedure.

	FOCO	THQ Opt via PSO
T50 (pre)	42.1 °C	42.4 °C
T50 (post)	42.7 °C	42.4 °C
T90 (pre)	41.1 °C	41.4 °C
T90 (post)	41.9 °C	41.7 °C
$\tau_{90}\;$(pre)	95%	96%
$\tau_{90}$ (post)	97%	97%

## Data Availability

Not applicable.
